# Innate Immunity and Biological Therapies for the Treatment of Sjögren’s Syndrome

**DOI:** 10.3390/ijms21239172

**Published:** 2020-12-01

**Authors:** Amrita Srivastava, Helen P. Makarenkova

**Affiliations:** Department of Molecular Medicine, The Scripps Research Institute, 10550 North Torrey Pines Rd., La Jolla, CA 92037, USA; amrisriv@scripps.edu

**Keywords:** Sjögren’s syndrome, autoimmune disease, B cell, T cell, macrophages, immune cells, immunotherapy, cytokines, dry eye, dry mouth, lacrimal gland, salivary glands, inflammation

## Abstract

Sjögren’s syndrome (SS) is a systemic autoimmune disorder affecting approximately 3% of the population in the United States. This disease has a female predilection and affects exocrine glands, including lacrimal and salivary glands. Dry eyes and dry mouths are the most common symptoms due to the loss of salivary and lacrimal gland function. Symptoms become more severe in secondary SS, where SS is present along with other autoimmune diseases like systemic lupus erythematosus, systemic sclerosis, or rheumatoid arthritis. It is known that aberrant activation of immune cells plays an important role in disease progression, however, the mechanism for these pathological changes in the immune system remains largely unknown. This review highlights the role of different immune cells in disease development, therapeutic treatments, and future strategies that are available to target various immune cells to cure the disease.

## 1. Introduction

Sjogren’s syndrome (SS) is an autoimmune disorder of the exocrine glands, including the lacrimal and salivary glands with a strong female predilection; women are affected 10–15 times more than men [1,2]. The disease is present among all age groups but generally starts between ages 40 to 60 years, affecting almost 3% of the population in the United States [3]. The main symptoms of the disease involve a reduction in saliva and tear secretion, leading to dry mouth (xerostomia/stomatitis sicca) and dry eyes (keratoconjunctivitis sicca). The disease can also spread to other organs, leading to various extra-glandular manifestations in the skin, gastrointestinal tracts, pulmonary system, liver, pancreas, kidneys, and nervous systems [4,5]. SS could be primary and secondary. Patients with primary SS show a loss of salivary and lacrimal gland function, while secondary SS develops in patients with other autoimmune diseases [6]. The major cause of illness in SS patients is due to fatigue and joint pain. One characteristic feature of this disease is hypergammaglobulinemia, defined by the presence of tissue-specific autoantibodies, the surge in the levels of immunoglobulins, circulating autoantibodies against ribonuclear proteins (anti-52 and 60-kDa Sjögren’s syndrome A; SS-A/Ro, anti-Sjögren’s syndrome B; SSB/La), cellular proteins like carbonic anhydrase II, cellular receptors (e.g., β-adrenergic, muscarinic cholinergic), secreted proteins, and detectable Rheumatoid Factor (RF) [7,8]. Production of anti-nuclear autoantibodies (ANAs) and interferon (IFN) are some of the additional features defining SS. Early diagnosis of patients with SS is very challenging and once the diagnosis is confirmed, there are no therapeutic treatments available to treat the disease etiology [9]. It was proposed that aberrant activation of immune cells is responsible for disease progression. However, the detailed mechanism of disease progression in the lacrimal and salivary glands are not determined [10]. This review focuses on innate immune-related events that occur during SS in the lacrimal and salivary glands, and functions of the different immune system components during disease progression, and therapeutic treatments and strategies that are available to target various immune cells to cure the disease.

## 2. Lacrimal and Salivary Glands Structure and Function

Lacrimal gland (LG) is an exocrine tubuloacinar gland that secretes the aqueous layer of the tear film. LG epithelium is composed of three major cell types—ductal, acinar, and myoepithelial cells (MECs). Acinar cells secrete the primary LG fluid, ductal cells modify the electrolyte composition of the primary LG fluid, before it exits the ducts and flows onto the ocular surface and MECs have a contractile function that helps to expel the secreted fluid from acinar cells [11]. 

The salivary glands are exocrine glands that produce saliva, a mixture of serous and mucous secretions containing water, proteins, glycoproteins, and electrolytes. The salivary glands also produce digestive enzymes that break down different nutrients. Humans have three paired major salivary glands—parotid, submandibular, and sublingual. The parotid glands are the largest salivary glands in humans [12]. Human and rodent parotid glands are composed of pure serous acini, while the human submandibular gland is a mixed gland composed of both serous and mucous acini. In rodents, the submandibular gland is composed of only the serous cells [13]. The acini of human and rodent sublingual glands are composed of mucous and serous cells [14]. 

## 3. Pathogenesis of Sjögren’s Syndrome

Alteration of glandular homeostasis is thought to be an initial event in SS, which happens before the onset of inflammation. Altered homeostasis can also activate the autoimmune response and inflammation. Exocrine dysfunction preceding inflammation was noticed in both mouse models and human patients. Experiments using the NOD mouse model, which is believed to have the same pathogenesis as humans, show an autoimmune phase preceded by non- or pre-immune phases [15,16]. In general, unusual proteolytic activity, high cell death, decrease in expression of the *EGF* gene, and changes in gene expression levels related to tissue homeostasis are observed before the autoimmune phase [17]. Increased nitric oxide (NO) production was also related to disease pathogenesis in SS patients. NO is generated by nitric oxide synthase (NOS), through the reaction of nitric oxide synthase (NOS) on l-arginine, which produces citrulline and NO [18]. An in vitro study involving mouse and human acinar cells obtained from salivary glands showed that chronic exposure to NO leads to the downregulation of their secretion [19]. Moreover, inducible nitric oxide synthase (iNOS) is a key regulator of the innate immune system [20]. NO is released by vascular endothelial cells and nerves [21] and can induce relaxation of the smooth muscle cells, including pericytes and myoepithelial cells. Decrease in contractile activity of myoepithelial cells leads to salivary and lacrimal gland dysfunction [22,23]. It was reported that in human salivary glands, NOS is localized in ductal epithelial cells [24]. In rat salivary glands, NOS isoforms were found in ductal and myoepithelial cells, while in the lacrimal glands, they localized in ductal and acinar cells. These findings suggest that nitric oxide can directly regulate secretion. In NOD mice, decrease in the salivary gland (submandibular and parotid) function precedes the autoimmune phase and happens in parallel to a decrease in nitric oxide synthase (NOS) activity. This was found prior to proinflammatory cytokine expression or formation of the lymphocytic infiltrations [25]. 

Further evidence related to the role of non-immune factors in secretory dysfunction was obtained from NOD–SCID mice, where the loss of acinar tissue (mainly due to increased protease activity) happens in the absence of inflammation [26]. It was shown that maintaining acinar cell polarity is crucial for the secretory function of the salivary and lacrimal gland in SS patients [27,28]. Rab3D and Rab8A proteins are required for the exocytosis function of the secretory pathway, and in SS patients it was noted that expression and distribution of the Rab3D protein changed and correlated well with the loss of cell polarity and secretory dysfunction [29]. Another factor that is independent of immune infiltration and linked to SS is high oxidative stress. High oxidative stress leads to overexpression of the reactive oxygen species (ROS) that further causes DNA damage and cell death, leading to a production of anti-DNA autoantibodies. High oxidative stress could lead to SS pathogenesis through ROS production, lipid membrane oxidation, and inflammatory process [30]. High oxidative stress also decreases lacrimal gland secretion by damaging the ocular surface epithelial cells [31], and it is inversely related to the levels of the antioxidant thioredoxin [32,33].

## 4. Innate Immune Cells in SS Disease 

**Figure 1:**
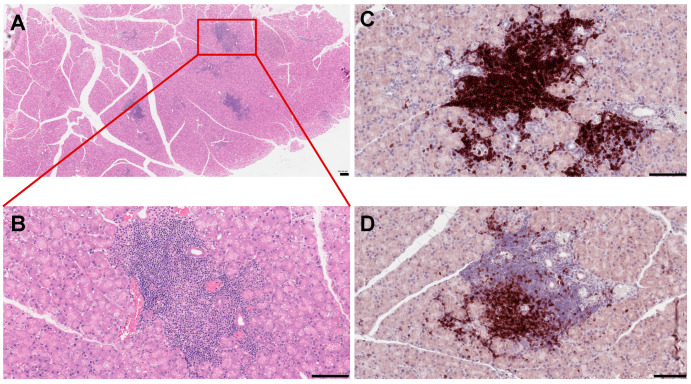
Histopathological features of mouse lacrimal gland at 3 months of age. (**A**) Histochemical staining of paraffin-embedded mouse lacrimal gland sections with hematoxyllin-eosin (H&E). (**B**) Higher magnification reveals severe infiltration of immune cells in the lacrimal gland. (**C**) Immunostaining of the NOD mouse lacrimal gland sections with the B220 antibody (B cell marker) (**D**) and CD3 antibody (a marker of T cells). Each scale bar is 100 μm.

Anomalous activation of the immune pathways leads to disease development in exocrine tissues and systemically to the destruction of epithelial cells (ECs) of the lacrimal and salivary glands. Similar to humans, the lacrimal gland of SS mouse models show periductal and perivascular loci of lymphocytic infiltrates (Figure 1), and loss of acinar and ductal cells, and hence loss of secretory function [34]. More severe destruction of the lacrimal gland was noticed with an increased duration of ocular disease [35]. The most common histological features of the salivary gland of SS patients include loss of tissue structure, acinar atrophy, and hyperplasia of the lining of the intraglandular ducts [36,37]. Several immune cells are implicated in SS progression. We recently reported that in several mouse models of SS, such as MRL/lpr, NOD (NOR/LtJ), and thrombospondin null (TSP1^−/−^) mice, the majority of cells forming the lymphocytic foci are B cells (Figure 1C,D) [38,39]. Infiltration of the gland involves CD4^+^ helper T (Th) cells, CD8^+^ cytotoxic T cells, B cells, plasma cells, macrophages, dendritic cells (DCs), and mast cells [40]. A more detailed analysis of male NOD mice showed the presence of B-cells (52.9%), CD4^+^ mature T helper cells (14.1%), CD8^+^ mature cytotoxic T cells (8%), NK cells (8.7%), macrophages (CD11b^+^ GR1^−^; 36.5%), and myeloid immunoregulatory cells (4.7%) in the lacrimal gland, indicating a serious inflammatory response [41]. The role of epithelial cells, DCs, T cells, B cells, natural killer T cells, and cytokines in disease development are well characterized and discussed. 

### 4.1. Epithelial Cells

The immunohistopathological analysis showed that diseased epithelium of the salivary and lacrimal gland plays an active role in the recruitment and activation of immune cells [1,42,43,44,45,46,47]. The epithelium releases autoantigens, Ro/SSA, and La/SSB, either by exosome or by apoptosis, thus, helping in an autoimmune response [48,49]. Co-stimulatory molecules present on salivary gland epithelial cells (SGEC), such as CD80, CD86, and CD40 mediate immune response by interacting with the immune cells to produce Th1 cytokine. Thus, the epithelium of salivary glands produces various chemokines including CXCL13 (C-X-C motif ligand 13), CCL17, CCL19, CCL21, and CCL22, which help to recruit the dendritic cells to the salivary gland [50]. Other chemokines like CXCL10 and CXCL9 help in T cells homing [51] and CXCL13 might help in B cell recruitment in the salivary gland, as an increase in expression of this chemokine leads to the development of the ectopic lymphoid structure that is mainly composed of B cells [52].

Epithelial cells also produce various cytokines (interleukins, IL) like IL-6, IL-7, IL-18, and IL-22 that are involved in T cell and B cell responses. IL-7 and IL-18 help in the Th1 response [53,54,55]. Epithelial cells of salivary glands also produce B cell-activating factor, BAFF after induction, which helps in B cell maturation, survival, and proliferation [56]. 

In addition, lacrimal gland epithelial cells express toll-like receptors (TLR), a type of glycoprotein receptors that are expressed in a variety of cell types, including epithelial cells [57,58]. TLRs recognize pathogen-associated molecular patterns on bacteria. TLR signaling plays an important role in epithelial cell function and survival. TLR-2, TLR-3, and TLR-4 upregulate the expression of Major Histocompatibility Complex-1 (MHC-1), CD45/ICAM-1 (Intercellular Adhesion Molecule 1), CD40, and CD95/Fas proteins, which play important roles in the adaptive immune system [59]. TLR-3 is expressed on the surface of activated salivary gland epithelial cells (SGECs) and its expression is significantly higher than other TLRs. TLR3 signaling leads to the death of epithelial cells through the upregulation of apoptotic proteins and the production of inflammatory cytokines [60]. The role of TLR3 signaling in the pathogenesis of SS was also confirmed in a mouse model where it not only reduced the salivary gland function but also upregulated type I IFN response and recruitment of B cells, dendritic cells, and NK cells in the target organ [61,62]. SS patients show increased expression of conjunctival ICAM-1 and associated lymphocyte-function-associated antigen-1 (LFA-1). ICAM-1 is a cell surface adhesion molecule and helps in the entry of lymphocytes to the site of inflammation [63].

### 4.2. B Cells (B Lyphocytes)

#### 4.2.1. Development and Function of B Cells

**Figure 2:**
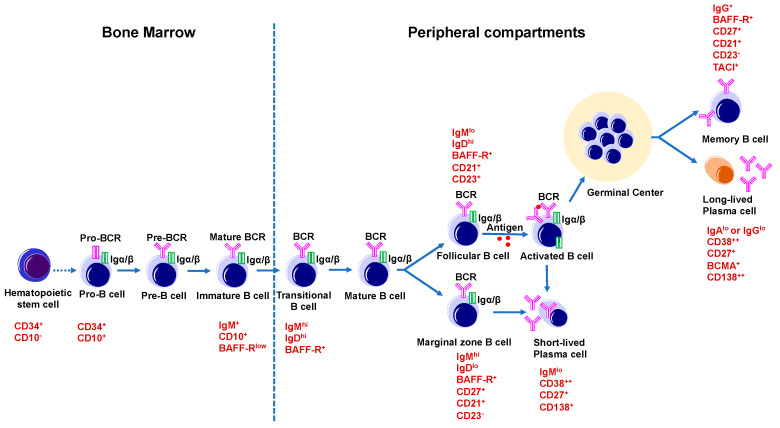
B cell development and maturation. B cells originate from hematopoietic stem cells in the bone marrow. Hematopoietic stem cell differentiates into common lymphoid progenitor cell, which give rise to a B lymphocyte progenitor that then differentiates into a Pro-B cell (CD34^+^CD10^+^), and a Pre-B cell, and then an immature B cell (IgM^+^CD10^+^BAFF-R^low^). In the spleen, immature B cells differentiate into naïve mature B cells. A small population of naïve mature B cells becomes marginal zone B cells (CD21^+^CD23^−^IgM^hi^IgD^lo^), whereas most of the naïve mature B cells develop into follicular B cells (CD21^+^CD23^+^IgM^lo^IgD^hi^). Later marginal zone B cells transform into short-lived plasma cells (CD38^++^CD138^+^) and follicular B cells, when encountering an antigen, and become activated B cells. These activated B cells take part in GC reactions in the secondary lymphoid organs like spleen and lymph nodes where they differentiate into long-lived, antibody-secreting plasma cells (CD38^++^CD138^++^BCMA^+^), or memory B cells (IgG^+^BAFF-R^+^CD27^+^CD21^+^CD23^−^).

B cells or B-lymphocytes have a specific function in the production of antibodies and play an important role in humoral immunity. In addition, B cells have diverse immunoglobulin (Ig) cell surface receptors that are able to identify different antigens [64]. Early B cell development [65] happens in the bone marrow from hematopoietic precursor cells (HSC), which differentiates into common lymphoid progenitor cells (CLP). Lymphoid progenitor cells start differentiating after receiving a signal from stromal B cells via cytokine secretion and cell–cell interaction. B cell development depends on the *Ig* gene rearrangement [66], leading to the production of antibodies to specific antigens. Thus, CLP’s differentiate into Pro-B cells (CD34^+^ /CD10^+^ /CD22^+^ /CD19^−^) that undergo *Ig* gene rearrangements and differentiate into Pre-B, which now express the B-lineage-specific cell surface molecule CD19. Pre-B cells gain heterogeneous CD20 expression and pre-BCR receptors and finally differentiate into immature B cells [67]. After this, immature B cell leaves the bone marrow and enters peripheral compartments like the spleen. The spleen is made up of various regions like the follicular region, which mainly consists of B cells. A marginal zone surrounds the follicular region that consists mainly of T, B cells, and macrophages. The red pulp in the spleen is a red blood cell disposal site, while the paracortical region mainly contains of T cells [68]. In the absence of MHC class II T cell help, immature B cells rapidly respond to T cell-independent type I antigens like lipopolysaccharides, and produce a quick antibody response. Additionally, immature B cells further develop into transitional B cells and then into mature B cells [65] (Figure 2). 

In general, most of these mature B cells are present in spleen and lymph nodes and can respond to foreign antigens in a T cell-dependent manner. These cells either differentiate into plasma cells or the germinal center (GC) cells. B cell receptors on specific B cells recognize a specific antigen and this antigen is processed inside the B cell and presented to CD4^+^ T cells or T helper cells on the major histocompatibility complex (MHC II) [69]. A cluster of differentiation 40 (CD40), found on antigen-presenting B cells, engages the CD40 ligand (CD40L) on CD4^+^ T-helper cells, and this interaction activates antigen-presenting B cells. After these interactions, T cells release cytokines like IL-4, which interact with B cells and might trigger some downstream signaling in B cells like hypermutation [70]. GC B cells, after interaction with pathogens, differentiate into long-lived plasma B cells or memory B cells [71]. These plasma B cells enter the bone marrow and play a crucial role in the defense mechanism against pathogens by secreting antibodies. Memory B cells have various subtypes. IgM^+^ memory B cells expand in the GC, while IgG^+^ memory B cells differentiate into plasmablasts (precursors of plasma cells) [72]. Marginal zone B cells and GC founder B cells are responsible for circulating a natural antibody pool and adaptive immunity. Antibodies secreted by marginal zone B cells act as a ligand for pathogens (Figure 2). B cells also play an important role in immune homeostasis by secreting various cytokines that control inflammation. Regulatory B cells (Bregs) produce anti-inflammatory cytokines like IL-10, IL-35, and TGF-β, and suppress the immune response. Bregs needs to be activated for cytokine production. There are two types of Bregs, innate, and adaptive. Innate Bregs produce IL-10 and IgM antibodies, while adaptive Bregs are activated by B-cell receptor (BCR) and CD40 co-stimulation [73,74]. In addition to immunosuppressive cytokines, B cells also produce proinflammatory cytokines like tumor necrosis factor (TNF-α) and IFN-γ, which direct the production of Ig isotypes such as IgG1 vs IgG4 [75].

#### 4.2.2. B Cells in Sjögren’s Syndrome 

B cells play multiple roles in SS pathogenesis. In addition to the production of autoantibodies and cytokines, B cells can also cause apoptosis of the epithelial cells in the salivary gland [76]. Activation of specific B cells is a crucial pathogenetic mechanism of SS. B cell activation is described by hypergammaglobulinemia, the formation of germinal center-like (GC-like) structures in the glandular tissue, and the production of RF and anti-Ro SSA/SSB antibodies [77,78].

Primary SS patients exhibit a reduction in the CD27^+^ memory B cells in peripheral blood, due to their migration or retention to the target organ [79,80,81,82,83,84]. In comparison to healthy controls, peripheral blood of SS patients contain a greater number of mature IgD^+^CD38^+^ and IgD^+^CD38^+^ cells (known as GC founder cells) [79]. These B cells express significantly higher levels of CD19, which might be helpful for BCR signaling in these cells. A higher number of these GC founder B cells might be explained by more frequent B cell activation in SS patients [79,85].

The lacrimal gland of SS patients shows lymphocytic infiltration involving clusters of B cells surrounded by scattered T cells [86,87]. Lymphocytic infiltration in the lacrimal gland might lead to the formation of GCs, and partial destruction of acini, leaving only a few interconnecting ductules [88]. There are few studies in the mice that demonstrate the importance of marginal zone B cells in SS disease development. For example, depletion of these cells in the *Tnfsf13b* transgenic mice (mice overexpressing BAFF), [89] NOD-*Aec1Aec2* mice (showing SS-like disease progression without diabetes) [90] and *Txlna*-transgenic mice (increased numbers of B1 cells and marginal zone B cells) [91,92] restored normal salivary gland function and showed normal histological features for lacrimal and salivary glands. Thus far, no human data on the roles of marginal zone B cells in SS patients are reported.

Another B cell population with a regulatory function is known as Bregs. These regulatory B cells produce immunosuppressive IL-10, control the proliferation of helper T cells (Th1), and regulatory T (Treg) cells [93,94]. In addition to IL-10, there are some other cytokines through which the regulatory functions of B cells can be carried out [93]. One of these cytokines is IL-35, which is part of the IL-12 family of cytokines. Two mouse studies were conducted to understand the regulatory function of IL-35. In one of the studies [95], it was shown that IL-35-induced B cells secreted IL-10 and IL-35. IL-35^+^ Bregs cells could inhibit Th1 and Th17 cells, and at the same time induce regulatory T cells (Tregs) proliferation, leading to a reduction in inflammation in mouse models of uveitis. This also suggests that Bregs and Tregs work synergistically to reduce inflammation. In another study [96], the protective role of IL-35^+^ producing B cells was seen in a case of experimental autoimmune encephalomyelitis, as mice in which B cells lack the expression of IL-35 could not recover from the T-cell-mediated encephalomyelitis. Later, it was described that there is a dysregulation of proinflammatory cytokine IL-12 and anti-inflammatory IL-35 in SS patients. Thus, in SS patients, serum levels of IL-12 were high, whereas the IL-35 level decreased [97]. Low disease symptoms were noticed in SS patients when IL-35 was high, indicating its protective role in the disease [98].

About 10–30% of SS patients also show an ectopic lymphoid structure in the epithelium of the salivary glands, which resembles the structure of a GC [88,99,100,101]. Like classical GCs, these GCs-like structures also participate in somatic hypermutation, BCR editing, and immunoglobulin class switching [102]. Thus, the GCs are involved in the pathogenesis of SS due to B cell activation and they might also participate in autoantibody production [103]. In mouse models, autoantibody-producing B cells are found to be at the margin of these GC-like structures [103], showing the involvement of these structures in the activation of autoimmune B cells. These structures are mainly formed by the expression of chemokines, the CXC-chemokine receptor 5 (CXCR5), and the CXC-chemokine ligand 13 (CXCL13). These molecules promote the development of GC-like structures by engaging the T follicular helper (Tfh) cells and B cells [104]. Thus, the Tfh cells upregulate CXCR5 and inducible T cell co-stimulator (ICOS). In response to the CXCL13 gradient, CXCR5 helps Tfh cells to migrate into B cell follicles, where they interact with B cells via the ICOS ligand (ICOSL) expressed by B cells. Tfh cells secrete a high amount of IL-21, which upregulates B cell lymphoma 6 (BCL6), and induces GC’s B cell differentiation. This leads to somatic hypermutation of the immunoglobulin genes, followed by isotype class-switching. Somatic hypermutation helps in generating antibody diversity. After the somatic hypermutation, B cells that show a high affinity towards antigen differentiate into high-affinity plasma cells and memory B cells [104] (Figure 2). 

BAFF cytokine can promote B cell maturation and differentiation. An elevated level of BAFF was seen in the SS patient’s serum, indicating its role in disease progression [105]. BAFF cytokine was also found in the salivary glands of SS patients. Generally, BAFF is produced mainly by myeloid cell lineages, such as monocytes, macrophages, and dendritic cells in healthy individuals, but in SS patients, epithelial cells and B and T cells might also secrete BAFF, demonstrating the role of epithelial cells in SS [106,107].

### 4.3. T Cells (T Lymphocytes)

#### 4.3.1. T Cell Development and Function

**Figure 3:**
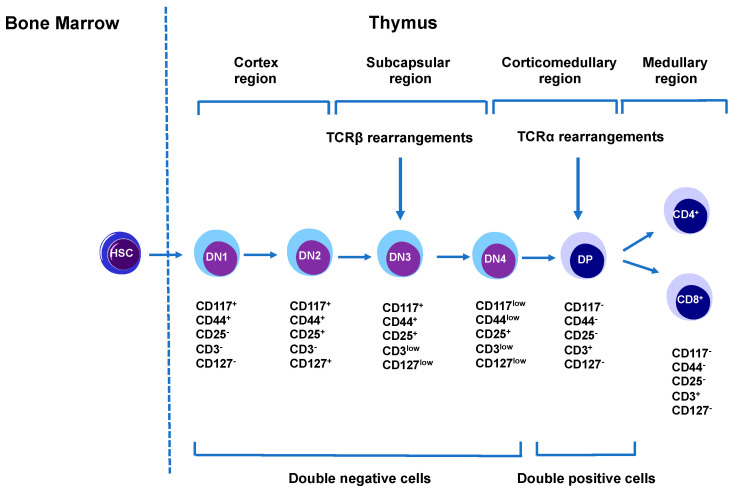
T cell development and maturation. T cells originate from hematopoietic stem cells in the bone marrow. These cells then migrate to thymus where they first develop into double-negative thymocytes (DN1-DN4). These thymocytes can be differentiated based on CD117, CD44, CD25, CD3, and CD 127 markers. DN4 then differentiates into double-positive (DP, CD4^+^CD8^+^) thymocytes after pre-TCR signaling. These DP’s differentiate into single-positive thymocytes as CD4^+^ and CD8^+^ cells.

T cells play a vital role in cell-mediated immunity. T cells are derived from the hematopoietic stem cells (HSC) in the bone marrow. T cell development takes place in the thymus, which is made up of several regions [108]. The subcapsular region of the thymus mainly consists of thymic epithelial cells (cTEC) (Figure 3). The cortex region is composed of cTEC, fibroblasts, and macrophages. The corticomedullary region (junction between cortex and medulla) contains endothelial cells that are responsible for the entry and exit of thymocytes to and from the blood, while the medullary region is composed of medullary thymic epithelial cells (mTECs) and dendritic cells (DC) [108]. HSCs firstly differentiate into double-negative (DN) cells. These are called double-negative as they lack the expression of both co-receptors CD4 and CD8. There are four populations of DN cells from DN1-4 that can be identified based on differential expression of CD25, CD44, and CD117 [109]. Double-negative 1 (DN1) cells are present in the corticomedullary junction of the adult thymus, where Notch 1 signaling plays a crucial checkpoint in T cell lineage commitment [110]. DN1 cells after leaving the corticomedullary junction, travel through the cortex and enter the subcapsular zone, where they interact with cTECs and fibroblasts and finally differentiate into DN2 subtypes. T cell receptors, TCRγ, TCRδ, and TCRβ gene rearrangements start at this stage. Within the subcapsular region, DN2 cells differentiate into DN3 cells. DN3 thymocytes rearrange their DNA at β, γ, and δ loci to express functional TCR chains. An important checkpoint known as β-selection takes place at this stage, via the transition of DN3a (CD27 low) to DN3b (CD27 high). Cells that rearranged their TCR-β chain locus are selected in this process [111]. In the subcapsular zone, the thymocytes continue to develop into the DN4 stage. After the pre-TCR signaling, thymocytes upregulate CD4 and CD8, hence attaining the double-positive (DP) stage. DP thymocytes interact with self-peptide MHC complexes and undergo a positive selection. These thymocytes now express self-MHC restricted TCR [112]. After this, these thymocytes transform into a single positive CD4 or CD8 lineage (Figure 3). Now, these single positive thymocytes migrate to the medulla, where they are screened for their reactivity to self-antigens, thus, removing the auto-antigen reactive T cells. These cells now attain a higher level of the sphingosine-1-phosphate receptor 1, which helps the cells exit from the thymus into circulation, where the ligands for this receptor are present in high concentrations [113], T cells act as a central regulator of the immune response and modulate other immune cell functions. There are various types of T cells and they play different roles in the immune system [114]. CD4^+^ T cells help in the maturation of B cells into plasma cells and memory B cells [115]. They also help in the activation of cytotoxic T cells and macrophages. These cells become activated when they recognize antigen fragments presented by antigen-presenting cells like B, dendritic cells, macrophages, and secrete cytokines [116]. Cytotoxic CD8^+^ T cells kill the pathogens like viruses and bacteria through three mechanisms. The first mechanism involves cytokines like IFN-γ and TNF-α, and these cytokines show anti-viral and anti-microbial properties. The second mechanism involves cytotoxic granules known as granzymes. Granzymes are serine proteases that contain two different proteins—perforin and granzyme. Initially, perforin creates a hole in the target cell membrane, and then granzyme enters the cell through the hole and cleaves the proteins, thus, stopping viral protein production and leading to apoptosis of the cell. The third mechanism involves Fas and Fas Ligand (FasL) interaction [117]. Activated cytotoxic T cells express Fas ligands on their surface, which binds to the Fas receptors present on the target cells. This interaction leads to the activation of the caspase cascade, resulting in apoptosis of the target cell [118]. Memory T cells are the antigen-specific cells, which when encountering an antigen that is associated with a previous infection or pathogen, quickly expand to a large number of effector T cells, thus, providing a quick response [119]. Another T cell type that is very crucial for immune response is Tregs. Tregs have two main roles. After the end of an immune response, they stop T cell-mediated immunity. They also suppress autoreactive T cells which in some way escape the negative selection process in the thymus [120]. Natural Killer T cells (NKT) recognize glycolipid antigens, and upon activation release cytokines to help the immune system. They also have cytotoxic properties [121].

#### 4.3.2. T Cells in Sjögren’s Syndrome

It is reported that in the early stages of the disease, T cells are the major infiltrating cells in lacrimal, and salivary glands, and among these cells, CD4^+^ T cells are the most common [63,122]. These CD4^+^ T cells have an activated phenotype, as demonstrated by Human Leukocyte Antigen isotype (HLA-DR and HLA-DQ) immunoreactivity [63]. T cells are responsible for the loss of self-tolerance in SS patients and secrete many proinflammatory cytokines like IFN-γ, IL-17, and IL-21 [123,124]. The pathogenic role of CD4^+^ T cells was also demonstrated in lacrimal keratoconjunctivitis in T cell-deficient nude mice [125]. Activation of T cells either by a viral infection or environmental factors leads to T cells with Th1 and Th2 phenotypes. Th17 and Th21 helper T cells provide a stimulus for B lymphocytes. T cells can also lead to the destruction of the gland through cytotoxicity [126]. CD8^+^ T killer cells that infiltrate the lacrimal gland display a cytotoxic phenotype and cause epithelial cell damage (reported in the NOD mice) [127]. These cytotoxic T cells have an activated phenotype and they produce inflammatory cytokines and promote dacryoadenitis even in the absence of CD4^+^ T cells, suggesting their pathogenic role in disease development in the lacrimal gland [127].

#### 4.3.3. Th1 and Th2 Cells

Ductal epithelial cells in the salivary glands secrete proinflammatory chemokines such as chemokine (C-X-C motif) ligand-9 (CXCL9) and chemokine (C-X-C motif) ligand-10 (CXCL10). These chemokines act as a ligand for the chemokine receptor—CXCR3, which is present on Th1 cells, and attract these cells to the epithelium [51]. Th1 cells largely produce IFN-γ and TNF-α. These cytokines activate macrophages, NK cells, and CD8^+^ T cells, and maintain cell-mediated immunity [128]. IFN-γ also affects salivary gland homeostasis [129]. The expression of the IFN-γ receptor was significantly higher in patients with SS with aqueous tear deficiency [130]. Th1 cells also secrete IL-18 that is present in CD68^+^ macrophages and the salivary gland’s ductal and acinar cells [54,131,132]. Higher levels of IL-18 were noticed in the sera and saliva of SS patients and NOD mice [133].

Th2 cells secrete IL-4, IL-5, and IL-13 cytokines and hence regulate humoral immunity by activation of B lymphocytes [134]. IL-4 is a very critical cytokine since a knockout of *IL4* in NOD and NOD.B10-H2b mice restored the salivary gland function. While these mice fail to develop exocrine gland dysfunction after mutation of IL4, they still exhibit pre-clinical manifestations of SS-like disease and glandular leucocyte infiltrations [134,135].

#### 4.3.4. Th17 Cells

Th17 cells are a subset of CD4^+^ memory effector T cells that secrete IL-17A and IL-17F, after stimulus from TGF-β and IL-6 [136]. IL-17A and IL-17F induce an inflammatory response that leads to the production of potent proinflammatory cytokines [137]. It was noted that the expression of IL-17 in the minor salivary glands of SS patients correlated with the severity of inflammation [138]. In SS patients, the minor salivary glands also increased the IL-17 protein and mRNA, as compared to healthy individuals [131,139,140]. Th17 cells also produce IL-21 and IL-22 [141]. IL-21 secreting the Th17 cells migrate to the salivary gland with the help of the gut-homing chemokine receptor CCR9. In the absence of IL-6, IL-21 helps in Th17 cell differentiation. IL-21 is also involved in the upregulation of Th17 cell-specific lineage transcription factors, and maintenance and stabilization of Th17 cells [142,143,144]. Differentiation of naïve B cells to plasma cells [145] and IgG isotype switching in B cells [146] are also facilitated by IL-21. It was shown that IgG isotype switching is required for the development of the SS in the NOD mouse model [135]. In addition, IL-21 is involved in GC formation by controlling the expression of Bcl-6, which is important for the GC response [147]. Thus, GC formation is related to the expression of IL-21 in labial salivary glands [148]. It is also reported that elevated levels of IL-21 leads to increased serum IgG levels and lymphocytic infiltrates in labial salivary glands [149]. Another Th17 cell cytokine, IL-22 is present at a higher level in SS patients and is directly related to hypergammaglobulinemia, hyposalivation, anti-SSA/SSB, and rheumatoid factor [150].

Murine models of SS showed the strongest evidence of IL17 and IL-17 producing cells (Th17) in SS disease [151]. No disease development was seen in IL-17 knockout mice. Elimination of IL-17 in B6.NOD-*Aec1Aec2* mice restored normal secretory function and reduced sialadenitis. This effect was more evident in females than in males. Further analysis of these mice revealed that IL-17 modulate Th2 cytokine as well as alter plasma cell and GC B cell populations in male and female mice. This study further revealed that IL-17 exhibits a sex difference in the process of disease [152]. 

#### 4.3.5. T Regulatory Cells (Tregs)

The role of Tregs in SS is not very clear. Tregs are identified by a high surface expression of the IL-2 receptor alpha chain (IL-2R*α*, CD25) and transcription factor Foxp3 (forkhead box P3), which is related to suppressor activity [153]. There are just a few reports about the increased number of activated memory Tregs in patients with autoimmune diseases, including SS. It was also found that the frequency of naïve Tregs remains unchanged [154]. In a group of SS patients with European League Against Rheumatism (EULAR) disease activity index (ESSDAI) > 5, frequency of memory Tregs is increased, whereas in other SS patients with less disease activity, no significant changes in memory Tregs compared to healthy individuals was observed. This finding suggests that disease progression is related to memory Tregs occurrence [126]. However, the functional role of these memory Tregs in SS patients is still unknown.

#### 4.3.6. Follicular Helper T Cells (Tfh) and Follicular Regulatory T Cells (Tfr)

The Tfh cells secrete IL-21, which helps in the regulation of T cell-dependent B cell responses [155]. IL-21 plays an important role in B cell activation and differentiation to plasma cells [156]. In blood and glandular tissues of SS patients, the frequency of Tfh cells is higher than in healthy individuals and generally increased the number of these cells related to several B cell-mediated autoimmune diseases [157,158,159,160,161]. The presence of the IL-21 protein and mRNA in the exocrine glands of primary SS patients further supported the occurrence of Tfh cells. It is unknown whether these cells are formed in endocrine tissues or they migrate from secondary lymphoid tissues. Production of these cells in salivary glands was evident from an in vitro experiment involving SGEC lines of primary SS patients, as these epithelial cells help in the differentiation of CD4^+^ naïve T cells into Tfh cells via upregulation of Inducible T-cell COStimulator ligand (ICOS-L) and IL-6 [162]. ICOS is mainly expressed on activated T cells and its ligand ICOS-L is expressed on antigen-presenting cells. ICOS belongs to CD28-superfamily costimulatory molecule and is important for Th2 cells [163]. It was also reported that in SS patients, a large number of circulating Tfh cells express CXCR3, which helps in the migration of these cells to the inflamed salivary glands, where CXCL10 is expressed [126]. 

Follicular regulatory T (Tfr) cells can control the proliferation of Tfh cells and B cell activation [164]. Tfr cells are present in high numbers in blood and minor salivary gland (MSG) tissues of SS patients [165,166]. Similar to humans, Tfr cells are present at the border of T cell and B cell zones in exocrine glands [167]. Due to their position outside the GC, Tfh cells can control GCs by interacting with B cells. 

### 4.4. Dendritic Cells (DCs) 

DCs are considered to be the most potent antigen-presenting cells and they play an important role in SS [168]. During the disease progression, the number of DCs decreases in peripheral blood but increases around epithelial cells in the salivary and lacrimal glands of SS patients [169,170]. Follicular DCs contribute to ectopic lymphoid neogenesis in SS because they are involved in T cell expansion and somatic hypermutation of B cells. A specific subset of DC, known as plasmacytoid DCs (pDCs), are associated with B cell infiltration and act as antigen-presenting cells (APCs). pDCs produce the majority of the type I interferons (IFN) and express high levels of TLR-7 and TLR-9, compared to conventional DCs. TLR-7 sense single-stranded RNA and TLR-9 unmethylated cytosine–phosphate–guanine (CpG) DNA, and this stimulation leads to the secretion of type-1 IFNs, IL-12, and other inflammatory cytokines that attract naïve CD8^+^ T cells to the T cell area [171]. In SS patients, pDC are present in lymphocytic foci of SS patients. pDCs cells initiate autoimmune responses and induce and maintain immune tolerance. Activated pDC presents autoantigen to T cells and lead to their priming and expansion of memory and naïve T cell population [172,173,174,175,176].

### 4.5. Natural Killers (NK) Cells 

NK cells are innate lymphocytes that express TLRs and communicate with DCs in maintaining adaptive immunity. In mice, NK cells are known to induce CD4^+^ T cell apoptosis by expressing the TNF-related apoptosis ligand. In humans, NK cells cause salivary gland inflammation. In SS patients, NK cells express a higher amount of activating receptor NCR3 (Natural Cytotoxicity Triggering Receptor 3) / NKp30, which binds to its ligand B7-H6 present, on dendritic cells and salivary gland epithelial cells (SGEC). Upon interacting with SGEC via its ligands, NK cells secrete IFN-γ, which plays a central role in the dysfunction of the salivary gland [177]. The number of NK cells in the peripheral blood of SS patients and their killing property is reduced, compared to healthy individuals [178].

### 4.6. Macrophages

Macrophages play a critical role in immune response and provide a link between innate and adaptive immunity. The total number of macrophages increases in both lacrimal and salivary glands of SS patients [179]. The pathogenic potential of salivary gland macrophages could be explained by the expression of IL-18 and CXCL13. The infiltration of macrophages into glandular tissue leads to the enlargement of the gland [132]. In the Aire-deficient mouse model of SS, macrophages play a functional role in dry eye disease as systemic depletion of macrophages leads to significant improvements in lacrimal gland structure and tear secretion in this mouse model [180]. It was reported that autoreactive CD4^+^ T cells play an important role in the infiltration of macrophages to the eye and lacrimal gland [180]. Tissue-resident macrophages also play a role in the development of SS. Thus, Ushio and coauthors showed that CD11b^high^ resident macrophages produce CCL22, which increases the production of CCR4 by T cells. CCR4 is the receptor of CCL22 on T cells and thus production of CCL22 by resident macrophages enhances the migratory activity of CD4^+^ T cells. They also showed that CCL22 might enhance the IFN-γ production by T cells, thereby suggesting its role in the impairment of local immune tolerance in the target organ of the SS model [181,182].

**Figure 4:**
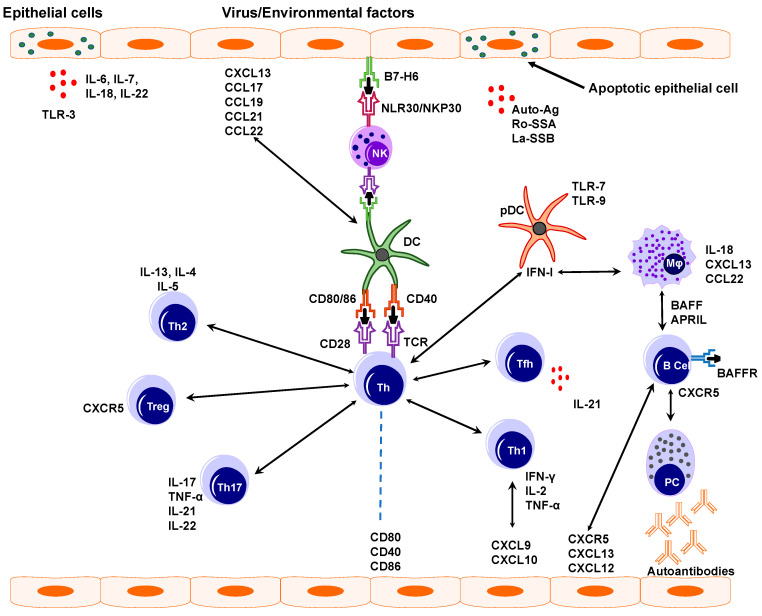
Pathogenesis of Sjögren’s syndrome. Virus or other environmental factors cause epithelial cell activation that will then express ligands, receptors, and various cytokines such as IL-6, IL-22, and chemokines like CXCL13. These further activate several other immune cells like natural killer cells (NK), dendritic cells (DCs), and macrophages (Mφ). Conventional dendritic cells interact with NK cells and T helper cells (Th) leading to increase IFN-γ production and tissue damage. IFN-1 and IFN-γ enhance BAFF production which leads to B and T cell activation. Activated B cells produce pathogenic autoantibodies.

In summary, multiple factors like genetic, environmental, and viral can induce damage to epithelial cells. The damaged epithelium then secretes various cytokines like interleukins, which further facilitate B and T cell response. After activation, B cells produce autoantibodies and several cytokines facilitating disease progression. Various B and T cell types are involved in SS pathogenesis. Dendritic cells and especially follicular dendritic cells contribute to T cell expansion and somatic hypermutation of B cells, increasing the dysfunction of glandular organs (Figure 4). Thus, various immune cells can contribute to the SS disease progression.

## 5. Biological Therapies for the Treatment of Sjögren’s Syndrome 

Symptomatic treatments of SS are the only treatments available thus far, no therapeutic treatment is available to cure the disease [183]. This could be due to the heterogeneity of the disease pathology. Several biological therapies reported in the literature are still in a clinical trial stage [184,185,186,187]. Among all of these therapies, B-cell-targeted therapy showed the most promising results in controlling the SS. Other therapies involving the targeting of T cells and cytokines are still in the early stages of the investigation [188]. Several diagnostic criteria of SS are reported in the literature [189]. The EULAR (see above) promoted a global collaboration to develop an SS disease activity index (ESSDAI) [190]. This activity index measures disease activity in patients with primary SS and is now used as a gold standard in clinical studies [191]. In addition, the EULAR Sjögren’s Syndrome Patient Reported Index (ESSPRI) [192], provided a questionnaire to the patients that helped to develop ESSDAI, and evaluated systemic complications [191]. Change in ESSDAI is often used as an outcome measure in clinical trials. Ocular dryness can be assessed by Schirmer tests and oral dryness through stimulated or unstimulated salivary flow rate [193]. Here, we discuss various clinical trials that showed encouraging results, and also consider some future targeting therapies for SS-related symptoms.

### 5.1. B Cell Targeting 

**Figure 5:**
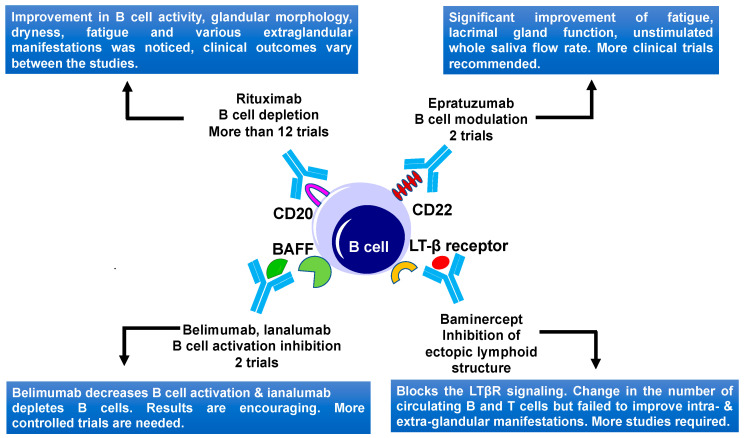
B cell-targeted therapies and their outcomes in primary Sjögren’s Syndrome. Current therapies include CD20, CD22, BAFF, and LTβ receptor targeting. BAFF, B-cell activating factor; LTβ, lymphotoxin β; and LTβR, lymphotoxin β receptor.

B cells play a central role in SS disease development and progression due to B cell hyperactivity, GC formation, and the production of SS autoantibodies [85]. Aberrant B cell activation might lead to extra glandular manifestations and changes of other serological characteristics in SS patients, including an increase in levels of free light chains and β2-microglobulin, rheumatoid factor, and hypergammaglobulinemia [7,194]. Ultimately unusual B cell activation might lead to the development of mucosa-associated lymphoid tissue (MALT) lymphoma, in some of the SS patients [195]. Several B cells targeting therapies including the B cell depletion and targeting of BCR signaling (Table 1, Figure 5) are reported up to date.

#### 5.1.1. CD20 Targeting

CD20 antigen, which was found on immature-B and mature B cells, was used as a target for B cell depletion therapy (Figure 5) [210]. Rituximab, a humanized anti-CD20 monoclonal antibody was used in several open-label studies to treat primary SS [211]. Binding of rituximab to CD20-expressing B cells leads to antibody-dependent cellular cytotoxicity, complement-mediated cytotoxicity, and apoptosis-mediated transient B cell depletion in peripheral blood, salivary glands, and other target tissues [212]. Recent studies suggested that rituximab modulates the exocrine gland immunological microenvironment [213]. Rituximab was reported to treat extranodal marginal zone B-cell lymphoma of the lacrimal gland [214]. Rituximab is the only anti-CD20 drug that was used to treat SS. Initial open-label studies on rituximab use in SS showed promising results, improving the fatigue and sicca symptoms [215]. Moreover, two small double-blind randomized studies showed a positive effect of rituximab treatment in SS (Figure 5). The first study involved 17 SS patients and showed an improvement of fatigue in the treated patients, as compared to the placebo group. No significant improvement was noticed in sicca symptoms (6 months study) [196]. A second study using rituximab involved 30 patients and showed significant improvement in the stimulated saliva flow rate, lacrimal gland function using the lissamine green test, sicca symptoms, and reduction in the number of extra glandular manifestations (that involve the kidneys, lungs, muscles, and the nervous system), including Raynaud’s phenomenon, tendomyalgia, and arthralgia. The functional improvements were seen between 12–36 weeks after rituximab treatment but all these positive effects were lost at week 48 in the rituximab-retreated patients [197].

In another study involving 78 SS patients with systemic involvement, treatment with rituximab led to the improvement of systemic manifestations like parotid swelling, pulmonary, and articular involvement, six months after the first treatment cycle [198]. Two other studies involving 28 and 41 SS patients focused on the evaluation of ESSDAI scores. Both of these studies showed an improvement in ESSDAI [199,200]. In the later study, patients received six courses of therapy and showed sustained improvement after a second dose of rituximab. A significant improvement in unstimulated salivary flow and Schirmer’s test was also noticed [200]. Later, two double-blind trials could not confirm these results due to a lack of homogeneity in patient characteristics and background medication that these patients used. The first study (known as Tolerance and Efficacy of Rituximab in Primary SS, TEARS) was done to assess the effectiveness and side effects of rituximab in 120 adults, with the recent onset of SS. Rituximab treatment failed to reach a primary endpoint like improvement of 30 mm in 2 of 4 visual analog scales (VAS), including primary activity, fatigue, pain, and dryness, at week 24. There was no effect on dryness measured by the Schirmer test for tear production or salivary gland inflammation [201]. Another trial named anti-B-cell therapy in patients with primary SS (TRACTISS) study, was performed in the UK, and involved 110 patients. Each patient was given two courses of rituximab or placebo to find out the extent to which rituximab treatment improved the symptoms of fatigue and oral dryness. There was no indication of improvement of symptoms related to oral dryness and fatigue. At the end of this study, there was no difference in the ESSDAI [202]. One more randomized trial including 133 primary SS patients with Ro antibodies focused on various outcome measures such as salivary and lacrimal flow rates, quality of life, reduction in fatigue, and oral dryness. This study showed improvement in unstimulated salivary flow, and no significant improvements in tear flow or any other outcomes [203].

Thus, rituximab treatment showed advantageous effects on B cell activity, dryness, fatigue, and several extra glandular manifestations in SS patients, although the effectiveness of rituximab was not the same for all patients (Figure 5). Discrepancies in these studies could be explained by the usage of different outcome measures. The primary outcome for two RCTs, TEARS, and TRACTISS was a change in subjective symptoms, such as a VAS score, but other subjective symptoms such as fatigue and sicca symptoms also contribute to the quality of life of SS patients. In this context, objective and subjective measurement of dryness should be equally considered. Future trials including composite endpoints that combine objective manifestations and subjective symptoms such as SS Responder Index (SSRI) [216] are needed to further evaluate the clinical response in SS patients. Though there is variation in the effectiveness of rituximab treatment in SS patients, this treatment still holds promise for patients with primary SS. Some other CD20 targeting humanized monoclonal antibodies, such as Ocrelizumab, Ofatumumab, and Veltuzumab were recently developed [217]. However, their efficacy and safety for treating the SS still needs to be examined.

#### 5.1.2. CD22 Targeting 

CD22 (cluster of differentiation-22) is a transmembrane protein that is highly expressed on the mature, and marginal-zone B cells [218]. CD22 regulates B cell function by CD19 and B cell antigen receptor (BCR) signaling and causing BCR-induced cell death. CD22 also regulates TLR signaling and controls the B cells survival in the peripheral organs [219]. CD22 might help in the entry of B cells to lacrimal and salivary glands of SS patients [220]. Epratuzumab, a humanized anti-CD22 monoclonal antibody, modulates the function of B cells instead of their depletion in the circulation (Figure 5) [221]. One open-label study including 16 SS patients (14 women and 2 men), who received 4 infusions with epratuzumab once every two weeks, were followed up to 6 months. The study focused on the main parameters involving the Schirmer-I test, unstimulated whole salivary flow, fatigue, erythrocyte sedimentation rate (ESR), and immunoglobulin G (IgG) measurement. This trial reported clinically significant responses in half of the patients, such as significant improvement of fatigue, lacrimal gland function, and unstimulated whole saliva flow rate at different time-points (Table 2, Figure 5) [204]. B cell level was decreased by 54% by week 6 and 39% at week 18, without affecting T cell levels and immunoglobulins. The overexpression of CD22 by peripheral B cells in these patients was also downregulated by epratuzumab for a minimum of 12 weeks, after ending the treatment, indicating potential promises of epratuzumab therapy [204]. Efficacy and safety of epratuzumab were also tested in 1584 patients sub-grouped in two populations, SLE patients with associated SS (EMBODY 1) and those without a diagnosis of associated SS (EMBODY 2). SLE patients with associated SS showed a faster reduction in B cells and improvement in SLE disease activity, compared to placebo [205]. 

#### 5.1.3. BAFF and APRIL Targeting

Another possible B cell targeting approach in SS patients involve interference with B cell activation. The B-cell activating factor axis contains two ligands, BAFF, and APRIL, and three receptors including B lymphocyte stimulator receptor 3 (BR3), B cell maturation antigen (BCMA), and TACI (transmembrane activator and calcium modulator and cyclophilin ligand interactor) [227]. BAFF (also called as B lymphocyte stimulator (BLyS)) is a member of the TNF-α family growth factors and is an essential cytokine for B cell activation, proliferation, and survival [228]. BAFF is upregulated by IFN-α, IFN-γ, and viruses [229]. In SS patients, increased levels of BAFF were noticed in serum, saliva, and salivary gland epithelial cells [105,230]. Excess BAFF levels mediate self-reactive B cell accumulation, which develops inflammation-associated autoimmune disease. Several biological therapies targeting BAFF were developed. Belimumab, a monoclonal antibody inhibits the B lymphocyte stimulator (BLyS)/BAFF (Figure 5). A phase II open-label study of Belimumab including 30 primary SS patients showed a reduction in parotid gland swelling and in levels of B cell activation biomarkers, indicating that this drug could decrease B cell activation. No change in unstimulated whole salivary flow or Schirmer’s test was noticed [206,231]. The results of this study are encouraging, and further controlled trials of this drug would be helpful in understanding the drug’s effects. In another clinical study, treatment with belimumab, followed by rituximab showed promising results [232]. Altogether, these studies might suggest that B cell activation and expansion happen at a different time of disease progression and that a combination of drugs blocking both events could have better outcomes as a therapy for SS (Figure 5).

Another monoclonal antibody ianalumab’s (VAY736) efficacy and safety was examined recently (Figure 5). This drug targets the BAFF receptor on the surface of B cells, leading to the lysis of B cells and blockade of BAFF receptor signaling [207]. Primary SS patients (n = 27) with (ESSDAI) ≥ 6 were divided into three groups. Six patients were given a single infusion of ianalumab at 3 mg/kg, 12 were given 10 mg/kg, and 9 a placebo. A positive therapeutic effect of ianalumab, supported by the ESSDAI and ESSPRI data, were observed in this study. However, the results of this study were variable, especially in parameters of saliva flow and ocular staining scores [207]. Nevertheless, this single-dose study suggested that B cell depletion by ianalumab might provide some therapeutic benefit to SS patients. A new study involving 190 primary SS patients was conducted to find out the dose-response curves of ianalumab. Patients were divided into 4 groups, three groups received ianalumab (5, 50, and 300 mg doses) and one group received placebo. The primary endpoint of this study was achieved. A statistically significant dose-response for ESSDAI was observed. The significant improvements in SS patients receiving 300 mg dose vs. the placebo group were also reported. This study is still ongoing [208].

Some other BAFF targeting antibodies are currently under investigation, mainly in SLE patients. These studies include treatments with soluble BAFF, recombinant fusion protein *atacicept*, which targets both Blys/BAFF and APRIL, *briobacept* (BR3-Fc), and *tabalumab*, which binds to membranes [233].

B cell receptor (BCR) signaling is important for B cell survival, proliferation, and function. This signaling mainly involves spleen tyrosine kinase, phosphatidylinositol 3-kinase delta isoform, and Bruton’s tyrosine kinase in BCR signal transduction [234,235]. It was noted that phosphatidylinositol 3-kinase delta isoform is activated in the glands of SS patients [236]. In another study, an increased level of Bruton’s tyrosine kinase was found in B cells of primary SS patients [237]. Therefore, targeting these kinases could be a valuable approach to treat primary SS patients. A small-molecule inhibitor of phosphatidylinositol 3-kinase delta isoform, UCB5857, was found effective in decreasing lymphocytic infiltration in a mouse model of ectopic lymphoneogenesis [236]. To test the efficacy and safety of UCB5857 in SS patients, a multicenter phase 2 study was started but stopped due to enrolment challenges (NCT02610543) [236].

#### 5.1.4. Lymphotoxin β Receptor Targeting

Lymphotoxin β receptor (LTβR) signaling helps in the maintenance and development of ectopic lymphoid organ structures formed by lymphoid infiltrates. LTβR binds to tumor necrosis factor superfamily member 14 (TNFSF14) and LTα/β heterotrimers. These LTα/β heterotrimers are present on the surface of mature B, T, and natural killer cells [238]. It was noticed that blocking the LTβR system inhibits the lymphocytic infiltration in the lymph nodes and in mucosal environments of mice [239]. In NOD mice, treatment with the lymphotoxin β receptor IgG fusion protein (LTβR-Ig) (also known as baminercept) prevents glandular inflammation and partly restores salivary flow. This effect is due to a combination of several factors, such as modulation of mRNA expression of various functional and disease-related genes in the lacrimal gland, a decrease in CXCL13 protein, removal of high endothelial venules, and decrease of lymphocyte uptake by the lacrimal gland [240]. To understand the clinical efficacy and the mechanism of baminercept action, a multicenter trial involving 52 SS patients was conducted. Patients were divided into two groups—one group received 100 mg of baminercept and another placebo. Treatment of SS patients with baminercept, showed statistically significant changes in the number of circulating B and T cells (Figure 5). However, intra-glandular and extra-glandular manifestations did not show any improvements [240]. Thus, the ESSDAI score shows no difference in patients treated with the baminercept, compared to the controls, despite blockage of LTβR signaling in the treated patients [209]. It is possible that blockage of LTβR signaling could still be beneficial for patients with early-stage disease. 

### 5.2. T Cells Targeting

**Figure 6:**
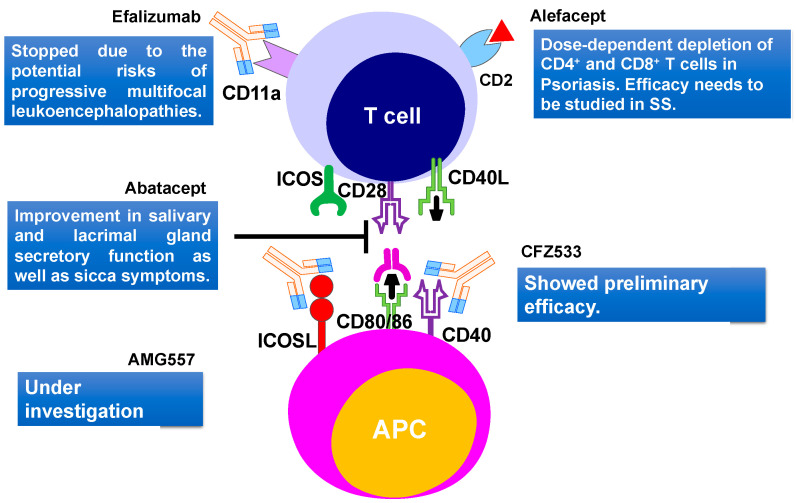
T cell-targeted therapies and their outcomes in primary Sjögren’s Syndrome. Current therapies include CD40, CD80/86, ICOSL, CD11a targeting. CD2, cluster of differentiation 2; CD40, Cluster of differentiation 40; CD28, Cluster of Differentiation 28; CD80, Cluster of differentiation 80; ICOS, inducible costimulatory; ICOSL, ICOS ligand.

It was proposed that T cells form a major part of the lymphocytic infiltrates in salivary and lacrimal glands, which mainly consists of CD4^+^ T cells at the early stage of the disease [241]. Interaction between activated CD4^+^ T cells and B cells, play an important role in B cell hyperactivity in SS patients. Therefore, targeting T and B cell interaction could be a powerful approach to treat the SS [126]. Abatacept is a human fusion molecule where the Fc region of human IgG1 is attached to human cytotoxic T lymphocyte antigen 4 (CTLA-4) protein (Figure 6). It prevents CD28-mediated T cell co-stimulatory signal, by blocking the crosstalk between the antigen-presenting cells and the T lymphocytes. Abatacept blocks the interaction of CD80/86 present on APC with the CD28 ligand, which is located on the surface of T cells, which is important for T cell proliferation and cytokine production [242]. In the open-label study, Alder and coauthors [222] demonstrated the effectiveness and safety of abatacept in patients with early stages of SS. Treatment of primary SS patients with abatacept led to a reduction of inflammation of the salivary gland and an increase in saliva production. Moreover, this treatment led to a significant increase in circulating B cells in the blood and a reduction of Treg frequency in the salivary glands. An increase in saliva production was comparable to rituximab therapy [222]. In another abatacept treatment study, 15 SS patients were treated with 8 doses of drugs. During the abatacept treatment, a significant reduction in ESSDAI, ESSPRI, rheumatoid factor, and IgG levels were noticed, but these factors increased again when the treatment stopped. However, the function of salivary and lacrimal glands did not change significantly during the treatment, whereas fatigue and quality of life improved significantly [223]. Abatacept was also found to be effective in a study involving 36 patients with SS associated with rheumatoid arthritis. Results showed an improvement in salivary and lacrimal gland secretory function, as well as the sicca symptoms [243]. Verstappen and coauthors [224] studied the effect of abatacept on the homeostasis of CD4^+^ T cell and B cell subsets, as well as on T-cell-dependent B cell hyperactivity in SS patients. They noted that abatacept reduces the number of circulating follicular helper T (Tfh) cells and Treg cells, whereas it had no effect on other CD4^+^ effector T cell subsets. Circulating CD4^+^ T cells decreased the expression of the activation marker ICOS, after treatment with abatacept. Lower ESSDAI scores of SS patients during treatment correlated well with reduced ICOS expression in the Tfh cells and decreased Tfh cell number [224]. To understand the effect of abatacept treatment on the histopathological changes of the parotid gland of SS patients, a pilot study involving 15 SS patients was performed [244]. No decrease in lymphocytic infiltrations, focus score, and number of CD20^+^ B cells were observed. However, a reduction of GCs was noticed with abatacept treatment, as the formation of GCs rely on co-stimulation of Tfh cells [244]. These studies revealed the importance of the early stages of SS treatment and hence the importance of early diagnostics. These studies also implied that early diagnosis can play a decisive role in choosing treatment strategies and improving the quality of life of SS patients.

A phase III clinical trial involving 80 SS patients with early and active stages of the disease evaluated the efficacy and safety of abatacept in primary SS patients. The study revealed no significant difference between the treated and the non-treated groups, based on the primary outcome ESSDAI score. Further studies need to be performed to conclude whether the abatacept treatment is beneficial for a specific group of SS patients [244].

Another humanized monoclonal antibody efalizumab, that targets the adhesion molecule CD11a subunit of leucocyte function-associated antigen-1 (LFA-1) was recently developed (Figure 6). The LFA-1 is thought to be involved in indirect interaction with T cell activation and reactivation [245]. This drug was initially approved for psoriasis and showed positive effects in TNF-α refractory patients [246]. The use of efalizumab trial in SS patients started in 2006 but stopped due to the potential risks of progressive multifocal leukoencephalopathies [247]. Efalizumab was withdrawn from the market in 2009. 

Cell surface glycoprotein, CD2 is present on T cells and is important in T cell adhesion and activation. A dimeric fusion protein Alefacept binds to the lymphocyte antigen CD2, inhibiting the leukocyte function-associated antigen-3 (LFA-3) and CD2 interaction interfering with the T lymphocyte activation. This protein was also used in psoriasis treatments [248]. A major concern for therapy was the dose-dependent depletion of CD4^+^ and CD8^+^ T cells [249]. A recent trial of alefacept involving 15 mg i.m. per week in type 1 diabetes showed encouraging results without major adverse effects [226]. Due to the similarity with the SS pathogenic mechanism involving memory T cells, this drug should also be studied further for the treatment of SS (Figure 6). Few other biological therapies like anti-CD40 treatment with CFZ533 (NCT02291029) [226] and anti-ICOSL treatment with AMG557 (NCT02334306) [226] are still under investigation (Figure 6).

The M3 muscarinic acetylcholine receptor (M3R) is a G protein-coupled receptor that is expressed in salivary and lacrimal glands and plays a central role in exocrine gland secretion [250,251]. Autoantibodies against lacrimal gland M3R, resulted in a primary, organ-specific dysfunction [252]. Thus, M3R could be a potential target for treating SS. M3R reactive T cells were found in the peripheral blood of SS patients. A study on Experimental Sialadenitis-like SS mice was performed using T cell epitopes of the M3R. In this study, cytokine production by M3R-reactive CD4^+^ T cells in response to M3R peptide was analyzed. Results showed the production of mainly interleukin-17 (IL-17) and interferon-γ (IFN-γ), in response to the M3R peptide. Altered peptide ligands for T cell epitopes designed for this study suppressed sialadenitis in vivo, through the induction of anergy and significantly suppressed IFN-γ production in vitro [253]. This indicates the potential of this strategy in controlling pathogenic T cell infiltrations in the glandular tissue of SS patients. 

As T cell-dependent B cell hyperactivity plays a crucial role in SS pathogenesis, targeting crosstalk between T and B cells could be a successful approach to treat SS. Therefore, treatments of the early stages of SS could be more beneficial than treatments of late stages. Various T cell-targeted therapies are listed in Table 2 and Figure 6.

### 5.3. Mesenchyme Stem Cells Transplantation

Mesenchymal stem cells (MSCs) are multipotent stromal cells with the ability of self-renewal and differentiation [254]. They are derived either from bone marrow (BMMSCs), umbilical cords (UCMSCs), gingiva MSCs (GMSCs), or adipose tissues (ADMSCs). MSCs are able to modulate the immune response of various immune cells, including dendritic cells (DC), macrophages, natural killer T cells (NKT), and mast cells (MC), which might also affect the pathogenesis of SS [255]. It was shown that the treatment of SS patients with MSCs improves sialadenitis, mainly by causing a decrease in Th1, Th17, and Tfh cells, and an increase in the number of Tregs [256,257].

The Tfh cells play an important role in B cell maturation and differentiation. Umbilical MSCs could be used as a novel therapeutic approach (Table 3) in SS patients, due to the production of indoleamine 2,3-dioxygenase (IDO) [256]. IDO converts tryptophan into kynurenine, thereby suppressing the T cell proliferation, while inducing differentiation of naïve T cells to FoxP3+ T regs [256,260]. At the same time, Alunno and coauthors found that human UMSCs had no effect on T cells implicated in SS, but just prevent the proliferation of healthy T cells [258]. Though UMSCs have a great therapeutic potential, it is difficult to use them in the transplantation approach, due to the systemic immune response in the host. In this context, a microencapsulation technique that separates UMSCs and T cells was developed [258]. These microencapsulated UMSCs restore the Tregs/Th17 ratio and suppress SS T cell proliferation [258], indicating the importance of a drug delivery platform in modulating the immunomodulatory effects of UMSCs in SS. In order to understand the mechanism of human UMSCs and their effect on SS pathogenesis, UMSCs were administered to NOD mice, prophylactically and therapeutically [261]. Tregs upregulation was noted showing the potential of UMSCs in treating SS [261].

Moreover, it was shown that cultured human UMSCs have the potential to differentiate into salivary gland epithelial cells. Thus, BMSCs co-cultured with the salivary gland epithelial cells develop comparable cellular structures like tight junctions and secretory granules, and also showed an increase in various salivary gland genes such as aquaporin 5, E-cadherin, and *α*-amylase [262,263]. This suggests that BMSCs transplantation is a promising therapy to treat SS by replacing damaged salivary gland acinar cells. However, a relatively recent study in mice in which BMSCs were delivered systemically through an i.p. injection, showed that, despite a positive temporary effect of this treatment on the lacrimal gland function, BMSC did not engraft into the epithelial component of the gland [264]. Several other studies also reported the therapeutic benefits of MSCs, even though engraftment of these cells into the epithelial compartment of the lacrimal gland was not detected [265,266].

The IL-12 level is known to increase in many autoimmune diseases and thus could be a potential target for SS treatment. Bingyu and coauthors analyzed the effect of MSC transplantation on IL-12 production, through dendritic cells in 29 SS patients. They found that DCs from SS patients produced more IL-12 compared to the control patients. Upon MSC transplantation, a reduction in IL-12 and an increase in saliva flow was noticed. MSCs also increased the number of Tregs and downregulated Th17 and Tfh cells [259]. 

Another study involving 404 patients with different autoimmune diseases, like SLE, SS, and rheumatoid arthritis was performed. Patients received allogeneic mesenchyme stem cell infusions and were evaluated for adverse events, to make sure that MSCs are safe to use as a treatment of autoimmune disease [267]. This study suggests that MSC infusion is a safe therapy for patients with autoimmune diseases.

In summary, MSCs have a potent immune-modulatory function because they suppress Th1/Th17/Tfh cell responses and upregulate Tregs. They can also modulate the function of DC, macrophages, mast cells, and NK cells. Due to their effect on the adaptive and innate immune system, MSCs could be used as a potential therapeutic treatment option for some SS patients. However, additional clinical trials are necessary to further understand MSC’s therapeutic potential for SS patients.

### 5.4. Cytokines as a Therapeutic Target

**Figure 7:**
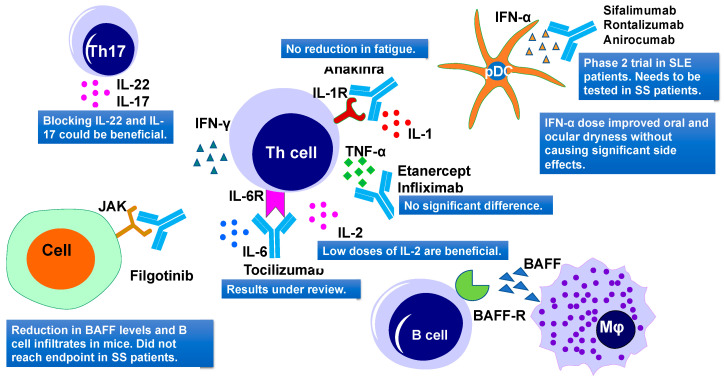
Cytokine-targeted therapies and their outcomes in primary Sjögren’s Syndrome. Current therapies include IL-1R, TNF-α, IL-6R, IL-2, JAK, IFN-α targeting. IL, interleukins; TNF-α, tumor necrosis factor–α; IFN-α, interferon α; and JAK, Janus kinase.

Cytokines constitute a complex signaling network and their dysregulation might lead to systemic or exocrine gland disorders. There are 38 interleukins (ILs) reported so far that could be used as potential therapeutic targets to treat several autoimmune diseases [268]. A list of cytokines that were used as a therapeutic target in SS is summarized in Table 4 and Figure 7. The majority of cytokine-targeted therapies in SS include the TNF family, IFN family, IL-1, IL-2, IL-6/12, IL-10, and IL-17 [269]. 

#### 5.4.1. TNF Family

TNF family members include TNF-α, TNF-β, CD40L, TRAIL, FasL, APRIL, and BAFF. TNF-α is the main component of SS pathogenesis and is secreted by CD4^+^ T cells, mononuclear cells, and epithelial cells. The impact of TNF-α targeting using infliximab was first reported in a small trial study involving 16 SS patients (Figure 7). Results showed a reduction in fatigue, joint pain, and sicca symptoms [270]. To confirm these results, Mariette and coauthors conducted a multicenter, randomized, double-blind, placebo-controlled trial (RCT) in 103 patients using TNF-α targeted drug infliximab, in 2004 [271]. However, this trial did not show any improvement in saliva flow rates, Schirmer test values, and salivary gland histology, but it reported improvements in patients’ fatigue (Figure 7). At the same time, severe adverse events were reported in the group treated with infliximab [271]. 

Another TNF-α targeting drug is Etanercept, which was used in two different trials, including one randomized controlled trial (RCT) and another prospective (Figure 7). In both studies, no significant difference was noticed between the treated and placebo groups for oral or ocular measures, Schirmer I test, and the saliva flow rate [272,273].

#### 5.4.2. IFN Family

Initially, interferons (IFN) were known as antiviral proteins but recently they were also recognized as regulators of non-viral processes, such as inflammation and immune modulation [279]. The IFN family includes multiple IFNs, such as type I IFN (IFN-I), type II IFN (IFN-γ), and type III IFN (IFN-III). IFN-I is a family of several related cytokines, which includes IFN-α, IFN-β, and IFN-ω [280]. Recent studies of IFN-I [281,282] and IFN-III [283] suggests their role in SS pathogenesis. IFN-I induces BAFF expression via interferon regulatory factors, IRF-I and IRFII, and downregulates BAFF expression via IRF4 and IRF8. IFN-I targeting could downregulate BAFF expression, leading to a less number of autoreactive B cells and decreased level of autoantibodies [284]. 

IFN-α was thought to increase the saliva flow rate in SS patients. To utilize its potential, Khurshudian and coauthors conducted a double-blind placebo-controlled study, involving 12 SS patients. Each day, patients received 3 doses of 150 IU of IFN-α for 24 weeks. It was found that IFN-α improved saliva production and relieved symptoms of xerostomia and xerophthalmia (Figure 7) [274]. Two other phase III clinical trials of IFN-α involving 497 SS patients showed similar results—improvement in oral and ocular dryness without causing significant side effects [275]. Several other trials targeting IFNs in RA and SLE are still ongoing [285]. IFNs bind to their receptors and activate Janus kinase (JAK)-signal transducer and the activator of transcription (STAT) pathway. JAK/STAT pathway is important for cytokine-dependent regulation of genes [286]. The safety and efficacy of topical ophthalmic tofacitinib, a Janus Kinase (JAK) inhibitor, was examined in treating dry eye disease by Liew and coauthors [276]. This study demonstrated improvement in some of the SS symptoms [276]. JAK1 inhibitor filgotinib application showed a reduction in BAFF levels and B cell infiltrates in the salivary glands of NOD/ShiLtJ mice (a mouse model of SS) [287]. The clinical trial of this drug in SS patients did not reach the primary endpoint (Figure 7). Several other monoclonal antibodies such as Sifalimumab (fully human, immunoglobulin G1 κ monoclonal antibody that binds to and neutralizes the majority of IFN-α subtype), [288] rontalizumab (a humanized IgG1 anti-interferon α (anti-IFN-α) monoclonal antibody) [289], and anirocumab (an anti-interferon-alpha receptor monoclonal antibody) [290] are in phase 2 clinical trials (Figure 7) in SLE patients, but their effectiveness needs to be evaluated in SS patients.

IFN-γ is secreted by NK cells, which activate the innate immune response, leading to damage of the lacrimal gland and the ocular surface [291,292]. IFN-γ level also increases in SS patients [293] and it could be a potential target in future therapies.

#### 5.4.3. IL-1 Family

Several cytokines like IL-1, IL-18, IL-33, IL-36, IL-37, and IL-38 are the key members of the IL-1 family. IL-1 cytokine could be a potential therapeutic target for treating SS, as it was found to be involved in SS pathogenesis [294]. A study on autoimmune-regulator (Aire)-deficient mice showed that IL-1 receptor knockdown reduced ocular surface keratinization, but did not reduce lacrimal gland lymphocytic infiltrations, suggesting that IL-1 receptor-targeted therapies could be beneficial only for treating ocular surface disease (Figure 7) [295]. Norheim and coauthors in 2012 used anakinra, an IL-1 receptor antagonist to treat fatigue in primary SS patients (Figure 7). However, they did not notice any significant reduction in fatigue by anakinra, as compared to patients treated with a placebo [277]. 

#### 5.4.4. IL-2 Family

The main members of the IL-2 family are IL-2, IL-4, IL-9, IL15, and IL-21. It was reported that IL-2 promotes Treg function, boosts T and B cell effector proliferation, and survival [296]. IL-2 also increases BAFF-stimulated cell viability/survival. Due to its contrasting immune-suppressing or stimulating roles, IL-2 can be used as a novel treatment in autoimmune diseases. Low IL-2 doses are found to be beneficial in autoimmunity, whereas high IL-2 doses increase antitumor immune responses [297]. As low doses of IL-2 promotes Treg function, this method was studied in type 1 diabetes [298] and systemic autoimmune diseases like SLE [299]. The short-term administration of low doses of IL-2 was evaluated in 190 patients with primary SS. It was noted that the numbers of Tregs were restored by IL-2 treatment, while no significant difference in Th17 cells was detected (Figure 7). Therefore, the normal Th17/Treg ratio was restored, which helped in controlling the disease progression in SS patients [300].

Another member of the IL-2 family, IL-21 also plays important roles in SS pathogenesis due to its effect on IFN-1 signaling and production of Tfh and Th17 cells [124]. In a recent SS study, a relatively higher level of IL-21 and IL-21 gene expression was noticed in the tears of SS patients compared to the controls [301]. It was also found that in SS patients, CD19^+^CD5^+^ B cells and invariant natural killer T cells, express an increased level of IL-21 receptor and IL-21, respectively, suggesting its role in regulating B cell functions [302].

#### 5.4.5. IL-6 and IL-12 Family

The IL-6/IL-12 family consists of various cytokines like IL-6, IL-11, IL-12, IL-23, IL-27, and IL-35. IL-6 level is increased in serum, saliva, and tears of SS patients. IL-6 is considered to be a promising therapeutic target, as it induces polarization of Tfh cells and participates in IL-21 induction [303]. Komai and coauthors and several other groups studied the effect of a humanized monoclonal antibody tocilizumab in SS patients (Figure 7). This antibody targets the IL-6 receptor and was found to be effective in two SS patients—one with refractory organizing pneumonia [278] and another with neuromyelitis optica spectrum disorder refractory to corticosteroids, plasma exchange, and cyclophosphamide [304]. In order to find out the efficacy of tocilizumab in primary SS, a randomized, double-blind, parallel, placebo-controlled trial was recently completed. The results of this trial are under review (NCT01782235) [304].

#### 5.4.6. IL-10 Family

The members of the IL-10 family are IL-10, IL-19, IL-20, IL-22, IL-24, and IL-26. It was reported that IL-22 expression is significantly increased in salivary glands of SS patients and its level is higher in serum. IL-22 is associated with hyposalivation and other SS symptoms, suggesting that it could be a potential therapeutic target to treat SS [55]. This idea further supported the study of the SS patients showing that hematopoietic cells have aberrant expression of IL-22 receptor, which is dependent on IL-18. Therefore, blocking the IL-18/IL-22 pathway could also be beneficial (Figure 7) [305].

Increased IL-17 levels were found in tears and serum of SS patients and several anti-IL-17A mAbs are currently being tested in various autoimmune diseases like RA and psoriasis (Figure 7) [306].

#### 5.4.7. Gene Therapy 

In addition to immune modulation, there is another approach to treat SS by engineering cells in a way to produce therapeutic proteins locally. This approach, known as gene therapy, avoids systemic immune suppression and causes minimum side effects. In this type of therapy, genes, RNAs, or regulatory micro RNAs are delivered to specific target cells, restoring the normal cellular function [307].

In an effort to understand the role of Th17 cells that secrete IL-17A in SS pathogenesis, Nguyen and coauthors [308] delivered adenoviral vectors expressing the IL17R:Fc fusion protein locally to salivary glands of the C57BL/6.NOD-Aec1Aec2 mice. Mice that received the IL17R:Fc expressing vector showed a decrease in CD4^+^IL-17^+^T cells in the spleen, lymphocytic infiltrations in the salivary glands, and an increase in salivary gland secretion, compared to mice treated with empty vectors [308].

Another gene therapy study aiming to understand the role of IL-27, a natural inhibitor of Th17 cell activity, in the SS mouse model was performed. Mice receiving recombinant serotype 2 adeno-associated viral (AAV2) vectors encoding IL-27, showed a decrease in disease progression [309]. Thus, inhibition of Th17 activity using gene therapies or immunomodulation could be used to improve disease outcomes in human patients.

It was reported that epithelia of salivary and lacrimal glands of SS patients and NOD mice express high levels of the bone morphogenetic protein 6 (BMP6) [310]. In mice, overexpression of BMP6 increases lymphocytic infiltrations and downregulates the salivary gland function [311]. 

In addition, Lai and coauthors [312] showed that BMP6 regulates expression of aquaporin 5. Water channel aquaporin 5 is expressed on the apical membrane of the secretory cells of salivary and lacrimal glands, and is vital for water secretion. One of the clinical trials demonstrated that the expression of AQP1 in the salivary glands after radiation-induced xerostomia could improve function of acinar cells and increase saliva flow [313]. In this study, aquaporin-1 (AQP1) was chosen because it permits passive transport of water along an osmotic gradient in a polarity-independent manner. In another study the AQP1 encoding adenoviral vector was delivered via cannulation into the submandibular salivary glands of the NOD-derived C57BL/6.NOD-Aec1/Aec2. This study suggests that AQP1 gene therapy might help in the restoration of fluid secretion from the salivary and lacrimal gland [312]. These approaches showed a great potential to treat SS pathogenesis and should be further investigated in SS patients.

Although gene therapy approach shows a real hope for SS patients, there are several concerns for using this type of therapy. The major concern is that the long-term gene expression requires the usage of constructs able to integrate into cellular genome. There is always a small possibility that such integration might disrupt normal function of endogenous genes. Recently, a novel gene-editing approach based on a bacterial CRISPR-associated protein-9 nuclease (Cas9) has been developed [314,315]. This gene editing approach shows significant promise, as it is highly specific and can be used for different types of genome editing, such as non-homologous end-joining, homology-directed repair, or single-base exchanges.

In the past few years, several laboratories tried CRISPR/Cas9 genome editing in mammalian preclinical models of eye disease [316]. A first clinical trial to evaluate safety and efficacy of the CRISPR/Cas9 gene editing therapy, to treat refractory viral keratitis is recently initiated.

## 6. Conclusions 

Early events in SS indicate that the central involvement of the innate immune system is crucial in the activation of early pathogenic mechanisms. Various cell types such as epithelial cells, T cells, B cells, dendritic, NK, and macrophages, participate in the activation of an immune response in SS. Therefore, it was suggested that targeting these cell types to modulate immune responses would improve disease outcomes. It was also evident that among all immune cells, B cells play a crucial role in the pathogenesis of SS due to B cell hyperactivity, GC, and autoantibody formation. Therefore, targeting B cells could be an attractive approach. B cells have several receptors, such as CD20, CD22, BAFF-R, and LT-β, which were used as targets to alleviate SS disease symptoms. Most of these studies targeted CD20, using rituximab as a drug that leads to the depletion of B cells. So far, more than 12 clinical studies were conducted to investigate the effectiveness of rituximab in SS patients. Generally, improvement in B cell activity, glandular morphology, dryness, and fatigue was noticed but there were some discrepancies in the results between the studies, which could be due to the employment of different outcome measures. It was also not clear which group of SS patients (at the early stage or the late stage of disease) would get more benefits from the rituximab treatment. Focused studies incorporating objective and subjective measurement of dryness would provide more insight into the effectiveness of the rituximab treatment. The focus should also be there to find out if rituximab had a more advantageous effect on early or late-stage SS patients. Another B cell target is CD22, and the Epratuzumab antibody was used to target it. This antibody acts by modulating the B cell function rather than depleting them, thereby acting as an immunomodulator. Two clinical studies showed that treatment with Epratuzumab improved fatigue, lacrimal gland function, and unstimulated whole saliva flow. A decrease in B cell level was also noticed, indicating the beneficial effect of Epratuzumab. Targeting BAFF and BAFF receptors with belimumab and ianalumab, showed positive results, and few ongoing studies are still investigating its potential for treating SS. Blocking the LTβ receptor with Baminercept, changes the number of B and T cells in circulation. However, glandular symptoms of primary SS did not show improvement, and the ESSDAI score also did not change. Therefore, it was suggested to try treating patients in the early stage of the disease, due to its effect on LTβR signaling.

During the initial phase of disease progression, T cells form first lymphocytic infiltrates, mainly consisting of CD4^+^T cells, and these activated T cells interact with B cells. Therefore, targeting T and B cell interaction seems to be a valuable approach to treat SS patients at an early stage of the disease. Abatacept improves the exocrine gland’s secretory function by blocking the crosstalk between B and T cells. However, a primary endpoint of the phase 3 trial study did not show significant improvement based on ESSDAI, indicating that future trials involving different groups of patients are needed to investigate the efficacy of abatacept treatment, in patients with an early stage of the disease. Another drug, alefacept, targeting the CD2 antigen present on T cell leads to the depletion of CD4^+^ and CD8^+^ T cells. Nonetheless, most of the T cell targeting strategies are still in the early phase of examination, and their efficacy needs to be further evaluated (Figure 6).

Inflammatory cytokines play an important role in epithelial homeostasis and regulate B and T cell function (Figure 7). Targeting TNF-α with etanercept and infliximab did not show any significant difference between the treated and placebo groups. In addition, several adverse effects were noticed for the SS patients treated with infliximab. It was noted that low doses of IL-2 and IFN-α are beneficial and they do not have many side effects. Tocilizumab, which targets IL-6 receptors, is still under investigation. As the level of IL-22, IL-17, and IFN-γ also increased during SS, therapies targeting these cytokines could be beneficial.

Thus, we can conclude that up to date B cell therapies are the most successful therapies, which additionally, were studied in more detail. At the same time, most of the current B cell therapies relied on the general depletion of B cells that might lead to a compromised immune system (Figure 5). Therefore, future development of therapies should focus on the targeting of the specific B cells responsible for disease development or progression. In this scenario, targeting marginal zone B cells could be a beneficial approach. Therefore, an approach that uses a combination of therapies targeting disease-specific T and B cells could be especially beneficial to treat patients with the SS disease. Alternatively, a sequential therapy, first T cell therapy followed by B cell therapy, might provide a better result.

Neutralizing pro-inflammatory cytokines or antagonizing their receptors could be a useful therapeutic strategy for some SS patients. This approach could be used as a complementary approach to other therapies. Unfortunately, the function of most cytokines is still poorly understood, due to the complexity and heterogeneity of the SS disease. We also think that detailed analysis of early stages of the SS disease and the mechanism of disease progression in patients and mouse models are necessary for the development of more personalized approaches to the successful treatment of SS patients.

## Figures and Tables

**Table 1:** B cell-targeted therapies in SS patients.

Drug	Target	Dose	No. of Pats	Type of Study	Efficacy	Side Effects	Refs
Rituximab	Chimeric mAb against CD20	Twice 1 g on days 1 and 15	17	Randomized, double-blind, Placebo-controlled pilot study	Improvement after 6 months, sicca symptoms did not improve	IRR, SSR	[196]
		1 g with an interval of 2 weeks or placebo	30	Prospective, single center, randomized, double-blind, placebo-controlled trial	Stimulated saliva flow rate and lacrimal gland function improvement	SSR	[197]
		375 mg/m^2^/week for 4 weeks or 1 g on days 1 and 15	78	Prospective study (AIR registry)	1st cycle efficacy in 47 patients (60 %) After 6 m ESSDAI decrease	IRR, SSR	[198]
		1 g with an interval of 15 days. patients received 6 courses of therapy	41	Prospective, multicenter, follow-up study	ESSDAI decrease. Reduction of infiltrate and GCs after treatment	No adverse effects	[199]
		Twice 1 g, 15 days apart	28	Prospective single-center study	ESSDAI and ESSPRI score improved.	Not reported	[200]
		Twice 1 g, two weeks apart	120	Randomized, double-blind, Placebo-controlled, parallel-group trial (TEARS)	No significant difference	Few patients had IRR	[201]
		two doses of rituximab (1 g) or placebo, two weeks apart	110	A randomized double-blind placebo-controlled clinical trial	No significant difference	Not reported	[202]
		Two courses of rituximab (1 g) at weeks 0, 2, 24, and 26 or placebo.	133	A multicenter, randomized, double-blind, placebo-controlled, parallel-group trial	No significant improvement in any outcome except unstimulated saliva flow	Few serious adverse events were reported but there were no deaths	[203]
Epratuzumab	Humanized anti-CD22 monoclonal antibody	4 infusions of 360 mg/m^2^ biweekly	16	An open-label phase I/II study	Improvements in fatigue. B-cell reduction, T cells did not change	Not reported	[204]
		600 mg every week, or epratuzumab 1200 mg every other week for 4 weeks	1584	Randomized, double-blind, placebo-controlled, multicenter studies	Disease activity in patients with SLE and associated SS showed improvements	Adverse events were comparable in the treated and placebo group	[205]
Belimumab	Human IgG1ʎ mAb targeting BAFF	10 mg/kg, monthly dose	30	Phase II open-label	In 60% of patients improvement in dryness, fatigue, and musculoskeletal pain	One patient develops pneumococcal meningitis	[206]
Ianalumab (VAY736)	a B cell-depleting, BAFF-R blocking, monoclonal antibody	single infusion at either 3 mg/kg, 10 mg/kg or placebo.	27	Double-blind, placebo-controlled, phase II, single-center study	Both doses lead to depletion of B cells for a long time	Moderate infusion related side effects	[207]
	BAFF-R	Monthly s.c. doses (5, 50, 300 mg) or placebo.	190	Phase 2b Study	Primary endpoint achieved, improvement for 300 mg dose	Safety profile looked good	[208]
Baminercept	Lymphotoxin-β receptor Fusion protein, reduces B cell infiltration	s.c. injections of 100 mg of baminercept every week for 24 weeks or placebo	52	Phase II multicenter, randomized, double-blind, placebo-controlled trial	No significant difference in ESSDAI, no difference in salivary gland secretion and ocular dryness	Higher incidence of liver toxicity	[209]

This table displays B cell targeted therapies for SS. The table displays drugs and drug’s dose, targets number of patients (Pats), study type, efficacy, and side effects. Abbreviations: mAb—monoclonal antibody, CD20—cluster of differentiation 20, IRR—infusion-related reaction, SSR—serum sickness-related, AIR airway intervention registry, ESSDAI—the EULAR Sjögren’s syndrome disease activity index, ESSPRI the EULAR SS patient reported index, MSG—minor salivary gland, TEARS -tolerance and efficacy of Rituximab in primary SS, SLE—Systemic lupus erythematosus, BAFF—B-cell activating factor.

**Table 2:** T cell targeted therapies in pSS patients.

Drug	Target	Dose	No. of Pats	Phase of Study	Efficacy	Side Effects	Refs
Abatacept	Anti-CD80/86, targets activation of T cells	8 doses of 500/750 mg, 2 weeks apart	11	A pilot study	CTLA-4 Ig treatment significantly reduces salivary gland inflammation, increases saliva production	No serious adverse effects	[222]
		8 infusions, first 3 were 2 weeks apart, then 4 weeks apart	15	Open-label study	Improvement in disease symptoms and fatigue.	No serious side effects or infections were seen	[223]
		~10 mg/kg by i.v. infusion on days 1, 15, and 29 and every 4 weeks thereafter for 24 weeks	15	Open-label study	Reduction of circulating Tfh cells and ICOS expression on T cells was noticed	Not reported	[224]
		125 mg s.c. once a week for 24 weeks or placebo	80	Single center, randomized, double-blind, phase 3 trial	ESSDAI no significant difference	Few serious adverse events reported	[225]
Alefacept	Anti-CD2 dimeric fusion protein	Two 12-week courses of 15 mg i.m. per week with a two-week interval or a placebo	73	Phase 2, double-blind, placebo-controlled	Lowered insulin usage and reduced hypoglycemic events	A severe drop in CD4^+^ and CD8^+^ T cells pose a major concern	[226]

This table displays T cell targeted therapies for SS. The table displays drugs, targets, drug’s dose, number of patients, study phase, efficacy, side effects, and references. *Abbreviations*: CD80/86-cluster of differentiation 80/86, CTLA-4Ig—cytotoxic lymphocyte-associated molecule-4 Immunoglobulin, ICOS inducible costimulatory molecule, i.m. intramuscular, CD2—cluster of differentiation 2, CD4—cluster of differentiation 4, and CD8—cluster of differentiation 8.

**Table 3:** Stem cells targeted therapies in pSS patients.

MSCs	Cell Number, Origin	Administration	Effect	Refs
UMSCs	1 × 10^6^ /Kg one dose	iv	Increase saliva flow, reduction in anti-SSA/Ro and anti-SSB/La antibodies	[257]
UMSCs	Human N/A	Coculture	Differentiation and proliferation of Tfh cells decreased	[256]
UMSCs microencapsulated	Human N/A	Coculture	Decrease in proliferation of T cells, and numbers of Th1, Th17; Treg increased	[258]
UMSCs	Human 1 × 10^6^ /Kg	iv	Reduced IL-12, decrease inTh17 and Tfh cells; Treg increased	[259]

This table displays SS therapies targeting mesenchymal stem cell (MSC). The table shows the origin of the MSCs, injected cell number, route of administration, effect of treatment, and references. *Abbreviations*: UMSCs—Umbilical cord-derived mesenchymal stem cell, iv—intravenous, SSA—Sjögren’s syndrome A antibodies, SSB—Sjögren’s syndrome B antibodies, Tfh—T follicular helper, Th1—T helper type 1, Th17—T helper 17, Treg—T regulatory cells, and IL-12—Interleukin-12.

**Table 4:** Cytokine-targeted therapies in pSS patients.

Drug	Cytokines	Target	Dose	No of Pats	Phase of Study	Efficacy	Side Effects	Refs
Infliximab	TNF family	TNF-α	3 mg/kg two weeks apart, three infusions	16	Phase II	Improvement in the visual analog score, fatigue, and dryness	No significant adverse events were seen	[270]
infliximab	TNF family	TNF-α	3 infusions of 5 mg/kg drug or placebo two weeks apart	103	Randomized, double-blind, placebo-controlled study	No significant differences	Severe adverse events reported in the infliximab group	[271]
Etanercept	TNF family	TNF-α	25 mg s.c. twice per week for 12 weeks	15	Pilot study	No increase in salivary or lacrimal gland function	Injection-site reactions occurring in about one-third of patients	[272,273]
IFN-α	IFN-α		150 IU of interferon-α 3 times a day for 24 weeks	12	Double-blind placebo-controlled	Improvement in symptoms of xerostomia and xerophthalmia	Well tolerated	[274]
IFN-α			150 IU of interferon-α 3 times a day for 24 weeks	497	2 Phase III clinical trials	Majority of symptoms improved	No significant adverse effect noted	[275]
Tofacitinib	IFN		0.0003–0.005% daily	327	Phase 1/2 prospective, randomized	Better patient-reported ocular tolerability	Well tolerated	[276]
Anakinra, a non-glycosylated recombinant version of the human IL-1 receptor antagonist, IL-lRa	IL-1	IL-1R blockade	100 mg/day or a placebo for 4 weeks	26	A double-blind, placebo-controlled parallel-group study	No significant changes	Two serious adverse events (SAE) were observed	[277]
Tocilizumab	IL-6	anti-IL-6 mAb	8 mg/kg	1	Case study	EULAR SS activity Index was stabilized at 4, CT scan and pulmonary function normalized	Treatment was well tolerated	[278]

This table displays the cytokine-targeted therapies for Sjögren’s syndrome reviewed in this article. The table displays cytokines and their target including drugs and their doses, number of patients, phase of study, efficacy, and side effects. Abbreviations: TNF Tumor necrosis factor, s.c. subcutaneous, IU International unit, IFN Interferon, IL-1 Interleukin-1, IL-1R Interleukin-1 receptor, and CT scan Computed tomography scan.
